# In This Issue

**Published:** 2001

## Introduction

Violence—whether personal violence, such as suicide; interpersonal violence, such as rape, homicide, or domestic abuse; or group violence, such as unruliness and riotous acts at sporting events—is a major concern to everyone. Alcohol abuse and alcoholism cause a significant number of severe—sometimes fatal—health, social, and economic problems for our country. That there is an association between alcohol use and all forms of violence has been known for many years. Understanding the association between the two could produce potent new ways to reduce their frequency and their consequences.

The National Institute of Justice (NIJ), a component of the United States Department of Justice, and the National Institute on Alcohol Abuse and Alcoholism (NIAAA) each has a long-standing interest in and commitment to funding research on violence and its causes, consequences, and prevention. This intersection of concern and interest in reducing alcohol-related violence has led over the years to a number of fruitful collaborations between NIAAA and NIJ. This issue of *Alcohol Research & Health* on alcohol-related violence is a fine example of such collaborations. In this issue, researchers supported by each agency share findings on a range of topics, including why some people may be more susceptible than others to committing or becoming victims of alcohol-related violence, how alcohol-use problems can lead to violence, and how violence itself may be a factor in the development of alcohol-use problems.

I would like to thank the NIJ staff for their invaluable assistance in identifying topics for articles and in helping to review manuscripts for this special issue of *Alcohol Research & Health*. We hope that this issue not only will highlight the problems associated with alcohol-related violence but also stimulate much-needed research attention to this subject.

Enoch Gordis, M.D., Director, National Institute on Alcohol Abuse and Alcoholism, National Institutes of Health

## ANTISOCIAL PERSONALITY DISORDER, ALCOHOL, AND AGGRESSION

Why are some people more likely than others to become aggressive after consuming alcohol? According to Drs. F. Gerard Moeller and Donald M. Dougherty, people with antisocial personality disorder (ASPD), a psychiatric condition characterized by a pervasive pattern of violent behavior and/or a general disregard for other people’s rights, may be particularly susceptible to alcohol-related aggression. In laboratory studies, people with ASPD were more aggressive after consuming alcohol than people without ASPD. Moreover, people with ASPD were more likely to abuse or become dependent on alcohol than people without the disorder. Even people without a diagnosis of ASPD, however, may respond to alcohol with increased aggressive behavior, especially if they show aggressive tendencies while sober. Various mechanisms, such as a person’s beliefs about alcohol’s effects and alcohol-related changes in brain function and brain chemistry, may play a role in alcohol-related aggression. (pp. 5–11)

## DIFFERENCES IN ALCOHOL-INDUCED AGGRESSION

Studying the mechanisms behind alcohol’s link to aggressive behavior in humans is difficult. Thus, researchers have relied on animal models to better define the alcohol-aggression relationship. Dr. J. Dee Higley reviews research in animals to show how individual differences in brain chemistry predict impulsivity, aggression, and alcohol-induced aggression. Dr. Higley describes a primate model specifically developed to study the mechanisms behind alcohol’s link to aggression and the characteristics that may affect this relationship. These characteristics appear to be associated with early rearing experiences and to remain stable throughout the animal’s life. (pp. 12–19)

## VICTIM AND OFFENDER SELF-REPORTS OF ALCOHOL INVOLVEMENT IN CRIME

Violent crime experienced an overall decline during the 1990s. Likewise, the number of violent crimes attributable to offenders who were drinking alcoholic beverages at the time of their offenses also decreased. Mr. Lawrence A. Greenfeld and Ms. Maureen A. Henneberg report on changes in alcohol-related violence evidenced by national surveys of crime victims and offenders conducted by the Bureau of Justice Statistics. According to the article’s authors, surveys of victims indicate that the rate of alcohol-related violent crime decreased more than the rate of non-alcohol-related violence. Surveys of some offenders also suggest that alcohol’s role in violence is decreasing. The decrease in alcohol-related violence is consistent with declines in other measures of alcohol use and misuse, including per capita alcohol consumption and alcohol involvement in traffic crashes. In contrast, violent offenders in State prisons are increasingly likely to report having used alcohol prior to their offense, possibly illustrating the effect of more severe sanctions for alcohol-related offenses. (pp. 20–31)

## COURT PROCEDURES FOR HANDLING INTOXICATED DRIVERS

Driving while intoxicated (DWI) is one of the most common criminal offenses associated with alcohol consumption, and many DWI offenders continue to drive intoxicated after they have been apprehended for the first time. To reduce this recidivism and deter DWI offenses in the first place, the courts have developed numerous sanctions. Drs. Robert B. Voas and Deborah A. Fisher provide an overview of these measures—including punitive sanctions (e.g., fines and incarceration), rehabilitative sanctions (e.g., alcoholism treatment), and incapacitating sanctions (e.g., license suspensions and ignition interlocks)—as well as review studies that have assessed the effectiveness of these measures. The authors also describe the various stages in the judicial process during which judges can implement those sanctions to maximize their effectiveness in reducing DWI recidivism. (pp. 32–42)

## ALCOHOL AND SEXUAL ASSAULT

Approximately one-half of all cases of sexual assault and rape involve alcohol consumption by the perpetrator, the victim, or both. In at least 80 percent of sexual assaults, both the perpetrator and the victim know each other; however, *alcohol-involved* sexual assaults often occur among strangers or people who do not know each other well (e.g., acquaintances or casual dates). Dr. Antonia Abbey, Ms. Tina Zawacki, Mr. Philip O. Buck, Ms. A. Monique Clinton, and Dr. Pam McAuslan explore the pathways through which alcohol may contribute to sexual assault. For example, certain beliefs regarding alcohol’s effects, attitudes concerning women’s alcohol consumption, and a high level of alcohol consumption may increase men’s propensity to commit sexual assault. For sexual assaults that occur in the context of social interactions, alcohol may impair both the victim’s and the perpetrator’s ability to interpret correctly subtle cues indicating a sexual interest or lack thereof. (pp. 43–51)

## ALCOHOL ABUSE AS A RISK FACTOR FOR AND CONSEQUENCE OF CHILD ABUSE

Researchers have investigated the role of alcohol abuse as both a cause and a consequence of child abuse. Although one might assume intuitively that parental alcohol abuse would increase a child’s risk of experiencing physical or sexual abuse and neglect, the studies conducted to date do not unequivocally support this assumption. Conversely, studies consistently have found that childhood abuse and neglect frequently are associated with adult alcohol problems, at least among women. Drs. Cathy Spatz Widom with Susanne Hiller-Sturmhöfel review studies on the relationship between alcohol abuse and child abuse. These studies have used a variety of samples, including general population samples, people undergoing alcoholism treatment, and people with court-documented histories of child abuse and neglect. The studies found that various factors, such as the use of alcohol as a coping mechanism, antisocial behavior, and the presence of post-traumatic stress disorder, may mediate the relationship between childhood abuse and neglect and adult alcohol abuse. (pp. 52–57)

## ALCOHOL-RELATED INTIMATE PARTNER VIOLENCE AMONG WHITE, BLACK, AND HISPANIC COUPLES IN THE UNITED STATES

As with other forms of violence, alcohol appears to play an important role in intimate partner violence (IPV). Survey results indicate that IPV is more prevalent among ethnic minorities than among whites. Researchers have proposed several theories to explain why rates of IPV vary among ethnic groups in the United States. For example, one main theory suggests that the cultural differences among ethnic groups account for the variation in IPV rates. Another theory points to social structural differences among ethnic groups. Drs. Raul Caetano, John Schafer, and Carol B. Cunradi discuss both these and other theories as well as review results from a recent national survey on IPV and alcohol use among U.S. couples. The researchers use the survey results to examine the relationships among ethnicity, IPV rates, alcohol use, and alcohol-related problems. They also use census data to examine the influence of neighborhood characteristics on IPV. The authors suggest that the higher prevalence of IPV among ethnic minorities is related to risk factors associated with the individual, the type of relationship between partners, and factors in the environment. (pp. 58–65)

## ALCOHOL AND VIOLENCE IN THE LIVES OF GANG MEMBERS

Life within a gang includes two endemic features: violence and alcohol. Yet, according to Drs. Geoffrey P. Hunt and Karen Joe Laidler, to date, most researchers of gang behavior have focused on violence and its relationship to illicit drugs, largely neglecting the importance of alcohol in gang life. Drs. Hunt and Laidler, however, show the extent to which drinking is a pervasive feature of gang life and discuss the ways in which drinking leads to different types and settings of violent behavior. By better understanding the link between drinking and violence among youth gangs, steps can be taken to determine the social processes that occur in the development of violent behavior after drinking. (pp. 66–71)

## SELF-REPORTED ALCOHOL USE AND ABUSE BY ARRESTEES IN THE 1998 ARRESTEE DRUG ABUSE MONITORING PROGRAM

Surveys of arrestees about their alcohol and other drug use provide valuable data that can be used to examine the relationship between substance use and violence. Dr. Susan E. Martin, Dr. Kendall Bryant, and Ms. Nora Fitzgerald present data collected in the Arrestee Drug Abuse Monitoring (ADAM) program for 1998, including the use of alcohol and other drugs by offenders and the relationships between substance use and the offenders’ gender, race, age, and types of offenses. In addition, the authors compare the data with a previous report from that program to identify changes in offenders’ alcohol and other drug use over time. (pp. 72–79)

